# Retrieving Inland Reservoir Water Quality Parameters Using Landsat 8-9 OLI and Sentinel-2 MSI Sensors with Empirical Multivariate Regression

**DOI:** 10.3390/ijerph19137725

**Published:** 2022-06-23

**Authors:** Haobin Meng, Jing Zhang, Zhen Zheng

**Affiliations:** 1 Beijing Key Laboratory of Resource Environment and Geographic Information System, Capital Normal University, Beijing 100048, China; 2210901016@cnu.edu.cn; 2 Key Laboratory of 3D Information Acquisition and Application of Ministry of Education, Capital Normal University, Beijing 100048, China; 3 Beijing Laboratory of Water Resources Security, Beijing 100048, China; 4 Fuzhou Research Academy of Environmental Sciences, Fuzhou 350000, China; zzme110@126.com

**Keywords:** water quality parameters, chlorophyll-a, algal density, turbidity, empirical multivariate regression, Landsat, Sentinel, Shanmei Reservoir

## Abstract

Improving water quality is one of the top priorities in the global agenda endorsed by the United Nations. To ensure the achievement of this goal, governments have developed plans to continuously monitor the status of inland waters. Remote sensing provides a low-cost, high-frequency, and practical complement to monitoring systems that can cover a large area. However, it is crucial to evaluate the suitability of sensors for retrieving water quality parameters (WQPs), owing to differences in spatial and spectral sampling from different satellites. Taking Shanmei Reservoir in Fuzhou City, Fujian Province as a case study, this study collected and sorted the water quality data measured at the site in 2020 to 2022 and Landsat 8-9 OLI and Sentinel-2 MSI images, simulated the chlorophyll-a (Chl-a) concentration, algae density, and turbidity using empirical multivariate regression, and explored the relationship between different WQPs using correlation analysis and principal component analysis (PCA). The results showed that the fitting effect of Landsat OLI data was better than that of the Sentinel-2 MSI data. The coefficient of determination (*R*^2^) values of Chl-a, algal density, and turbidity simulated by Landsat OLI data were 0.70, 0.81, and 0.80, respectively. Furthermore, the parameters of its validation equation were also smaller than those of Sentinel MSI data. The spatial distribution of three key WQPs retrieved from Landsat OLI data shows their values were generally low, with the mean values of the Chl-a concentration, algal density, and turbidity being 4.25 μg/L, 4.11 × 10^6^ cells/L, and 1.86 NTU, respectively. However, from the end of February 2022, the values of the Chl-a concentration and algae density in the reservoir gradually increase, and the risk of water eutrophication also increases. Therefore, it is still necessary to pay continuous attention and formulate corresponding water quality management measures. The correlation analysis shows that the three key WQPs in this study have a high correlation with pH, water temperature (WT), and dissolved oxygen (DO). The results of PCA showed that pH, DO, Chl-a concentration, WT, TN, and COD_Mn_ were dominant in PC1, explaining 35.57% of the total variation, and conductivity, algal density, and WT were dominant in PC2, explaining 13.34% of the total variation. Therefore, the water quality of the Shanmei Reservoir can be better evaluated by measuring pH, conductivity, and WT at the monitoring station, or by establishing the regression fitting equations between DO, COD_Mn_, and TN. The regression algorithm used in this study can identify the most important water quality features in the Shanmei Reservoir, which can be used to monitor the nutritional status of the reservoir and provide a reference for other similar inland water bodies.

## 1. Introduction

Inland waters cover approximately 3% of the terrestrial surface of the Earth and have many important functions including providing ecosystem services such as hydroelectric power, flood protection, navigation, water supply, and fisheries [1,2]. It is estimated that approximately one in eight global citizens still do not have access to safe drinking water, although access has increased in recent years [3]. With the water demand in some countries likely to exceed the supply by 50%, nearly half the global population will face water scarcity by 2030 [4]. Therefore, water resource management is particularly important to ensure that there is a sufficient water quantity of adequate quality for multiple human uses by managing water resources.

Reservoirs are a distinct example of inland water bodies and are transitional systems between rivers and lakes formed by the damming of rivers. Reservoirs are also simple targets for waste disposal [5]. The biotic and abiotic variables of these functionally complex ecosystems undergo rapid changes owing to natural environmental changes, changes in watershed land cover and land use, and changes in water demand [6]. The construction and use of reservoirs change the hydrodynamics of rivers, with different impacts on terrestrial and aquatic systems [7]. With an increase in residence time, the effective utilization time of nutrients is prolonged, and water bodies become increasingly eutrophic [8]. Therefore, monitoring WQPs is crucial for maintaining the health of these water bodies.

WQPs are usually obtained using in situ sensor probes or by analyzing water samples collected in the field. These traditional methods are laborious, expensive, and have limited spatial coverage [9]. Remote sensing, with its advantages of broad spatial coverage and repetitive temporal coverage, can complement in situ measurements. Using remote sensing, maps showing the spatial distribution of WQPs can be generated at multiple time intervals for monitoring purposes. Therefore, remote sensing technology, which can simultaneously monitor large areas, has been widely used.

Since the 1960s, remote sensing techniques have been used to monitor aquatic environments by analyzing ocean colors under the assumption that Chl-a (a quantified proxy for phytoplankton biomass) and surface temperature can be estimated remotely [5,10]. Based on this, many researchers have used satellite sensors to evaluate WQPs with optically active parameters, such as total suspended matter, Chl-a concentration, turbidity, phytoplankton pigments, and color-dissolved organic matter (CDOM) [11,12,13]. However, estimating TN, TP, and COD concentrations in inland waters presents a great challenge. As the above parameters are not optically active at the sensed wavelengths [14], it is difficult to directly correlate remote sensing spectral characteristics with TN, TP, and COD concentrations [15], so most of the current studies using conventional remote sensing monitoring methods focus on WQPs with optical activity. In terms of WQPs retrieval, previous studies were based mainly on different correlation algorithms of empirical, semi-analytical, and matrix retrieval models. Semi-analytical models are based on radiative transfer theory and require bio-optical and empirical data to describe the relationship between the components of a water body and the equivalent surface reflectance that defines the upwelling radiance above and on the surface of the water [16,17]. There are three general types of semi-analytical models, one of which is the retrieval and optimization algorithm [18]. It uses a forward model to simulate spectra from multiple parameters and selects the set of parameters that minimize the chosen cost function as the solution [19]. If the forward model is linear and the cost function is the sum of the squares of the residuals, this is reduced to the linear matrix retrieval method [20]. However, owing to the lack of specific parameters, matrix retrieval methods are complicated and difficult to calibrate. Therefore, empirical algorithms are typically employed to retrieve and estimate WQPs [21,22].

The continuous development of remote sensing and geographic information science has significantly improved the efficiency of geographical feature analysis [23,24,25]. The increased frequency of image acquisition and advances in data processing capabilities have provided new opportunities for understanding complex inland water systems [26]. Remote sensing-based assessments and water monitoring may use the same methods for retrieval and prediction, but various sensors can be used for research. For example, the Moderate-Resolution Imaging Spectroradiometer (MODIS), Medium-Resolution Imaging Spectrometer (MERIS), MultiSpectral Instrument (MSI), and Operational Land Imager (OLI) can be used. These sensors are different in spatial, temporal, spectral, and radiometric resolutions, and several studies have been carried out to estimate WQPs using these sensors [27,28,29]. The applicability of WV-2 imagery with existing effective estimation methods from MERIS when estimating the Chl-a concentration in inland turbidity waters was verified for Guanting Reservoir, where the correlation analysis of the measured Chl-a concentration content and WV-2 imagery bands show that the bands of WV-2 sensitive to the Chl-a concentration are red edge, NIR 1, and NIR 2 [30]. A study on Araucanian lakes developed and validated empirical models to estimate turbidity values from Landsat images and determine the spatial distribution thereof [31]. Yashon et al. proposed an empirical multivariate regression model (EMRM) algorithmic approach for estimating the Chl-a concentration, total suspended solids (TSS), and turbidity associated with field laboratory measurements; the results showed that the algorithms developed are broadly able to discern the bio-optical quality of water in reservoirs, even if the absolute accuracy of the retrieval of the WQPs still requires improvements [17]. Using Landsat satellite images, 11 spectral indicators were calculated, and the correlation between the vegetation index and Chl-a concentration in different monitoring areas was established. The indicators with the best correlations were the normalized difference vegetation index (NDVI) and the green normalized difference vegetation index (GNDVI) [32].

Taking the Shanmei Reservoir in Fujian Province, China, as a case study, the aims of this work were to (1) describe the reflection characteristics of the water body in different bands of the Sentinel MSI and Landsat OLI data combined with the observed water quality data for the reservoir; (2) generate and validate empirical models for WQPs from the two satellite sensors, by comparing the size of validation parameters (MAE, MSE, RMSE), and select the remote sensing inversion model more suitable for the reservoir; (3) retrieve the Chl-a, algal density, and turbidity with optical activity according to the regression formula to understand the current status and changing characteristics of the water quality of the reservoir, and (4) explore the relationship between WQPs, and select the factors that have a greater impact on the water quality, so as to provide a reference for the rapid monitoring of the water quality of the reservoir in the future. Establishing simple models with high accuracy and known errors will facilitate rapid, accurate, and real-time evaluation of water quality using measured data and remote sensing techniques. We hope that this study can provide a reference for the further study of reservoir water quality, which is conducive to the monitoring and early warning of reservoir nutritional status, ensuring the safety of downstream people’s life and farmland water, and creating a better reservoir environment.

## 2. Materials and Methods

### 2.1. Overview of the Study Area

**Figure 1:**
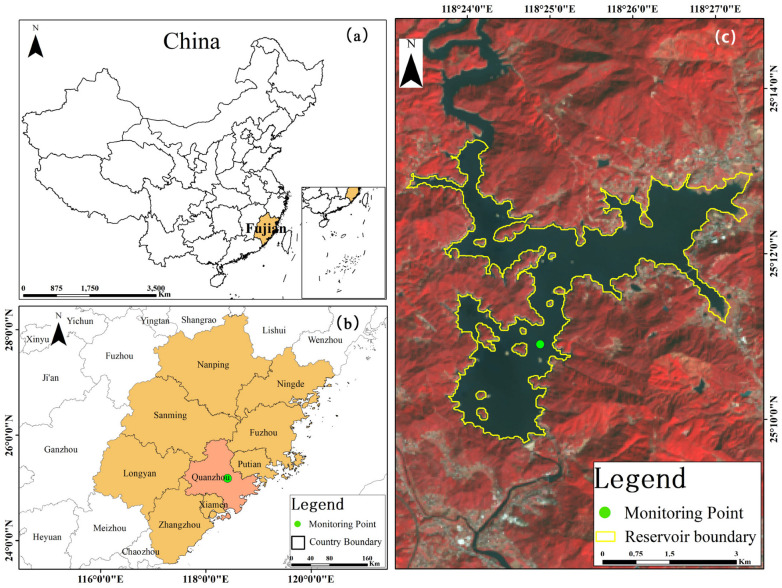
(**a**) Fujian Province in the yellow section is located on the southeastern coast of China, (**b**) Quanzhou City in the pink section is located at the eastern end of central Fujian Province, and (**c**) the boundary of Shanmei Reservoir. The green point is the location of the monitoring station.

The Shanmei Reservoir is in Quanzhou City (25°07′41″ N, 118°26′36″ E) in the middle reaches of the Dongxi River, a tributary of the Jinjiang River, one of the four major rivers in China (Figure 1). The reservoir provides water for 4 million people living downstream, supplies 43,300 hectares of farmland with irrigation water, and provides water to ensure the sustainable development of Quanzhou’s economy and society [33,34].

The Shanmei Reservoir has an area of 26 km^2^, a length of 12 km, a maximum width of 7 km, and a maximum depth of 50 m, with a total storage capacity of 655 million m^3^ and a normal water level of 96.48 m. In recent years, increasing attention has been paid to the water quality of Shanmei Reservoir. With the economic and social development of the catchment area, the reservoir has faced new or intensified challenges including (1) the increased pressure of eutrophication in the reservoir area, (2) the increased risk of seasonal algal blooms, and (3) TN functioning as a nutrient source. As a result, the optimization of the aquatic biological community structure and the reduction of the algal bloom risk have become key issues in the Shanmei Reservoir.

### 2.2. Data Collection

#### 2.2.1. In Situ Data

The in situ data were obtained from the national surface water quality automatic monitoring real-time data publishing system (http://106.37.208.243:8068/GJZ/Business/Publish/Main.html, (accessed on 2 November 2021)), which was issued by the Chinese Ministry of Environmental Protection on 1 July 2009 and released to the public. The main indicators included WT (°C), pH, DO (mg/L), conductivity (μS/cm), turbidity (nephelometric turbidity unit, NTU), COD_Mn_ (mg/L), ammonia nitrogen (NH_3_-N, mg/L), TN (mg/L), TP (mg/L), Chl-a concentration (mg/L), and algal density (cells/L). The monitoring frequency is once every four hours, and the monitoring data were dynamically released six times a day (0:00, 4:00, 8:00, 12:00, 16:00, and 20:00). The methods of measuring WQPs are detailed in technical specifications for automatic monitoring of surface water (HJ 915-2017) issued by the Ministry of Ecology and Environment of the People’s Republic of China (https://www.mee.gov.cn/ywgz/fgbz/bz/bzwb/jcffbz/201801/t20180108_429283.shtml, (accessed on 2 November 2021)). This study used Chl-a concentration, turbidity, and algal density data from 1 November 2020 to 26 February 2022, and the monitoring point was located at 118°24′52″ E, 25°10′54″ N. According to the introduction of Landsat 8-9 satellite and Sentinel-2 satellite, the acquisition time of both satellites is approximately 10:30 local time. Therefore, the WQPs data at 8:00 and 12:00 are selected for averaging, and the single remote sensing image pixel where the water quality monitoring station is located is selected to match the water quality data.

#### 2.2.2. Landsat 8-9 OLI Data

Landsat-8 and Landsat-9 satellites were launched on 11 February 2013 and 27 September 2021, respectively. The two satellites have 11 frequency bands, and the global coverage can be realized every 8 days after the data combination. In December 2020, the USGS reprocessed archived Landsat data and released a new collection, Collection 2, which introduced surface reflectance and surface temperature Level-2 products, implemented improved ground control and elevation datasets, brought several geometric and radiometric calibration enhancements, and improved the atmospheric correction algorithm. We selected 14 Landsat 8-9 Collection 2 Level 2 products (https://earthexplorer.usgs.gov, accessed on 24 November 2021), which had been geometrically corrected, radiometrically calibrated, and atmospherically corrected, and can be used directly after processing according to the following formula. Table 1 presents the dates of selected Landsat 8-9 images.

(1) $$R_{rs(\lambda )}=0.0000275\times Pixle\;Value-0.2$$

#### 2.2.3. Sentinel-2 MSI Data

Sentinel-2A and 2B satellites are high-resolution multispectral imaging satellites. Both satellites have a 10-day revisit period, and when combined, they can achieve global coverage every 5 days. For Sentinel-2A and -2B data, the L1C multispectral data released by the European Space Agency (ESA) is only an orthophoto image after geometric fine correction, which also requires radiometric calibration and atmospheric correction. The current mainstream software is Sen2Cor (a processor for Sentinel-2 Level 2A product generation and formatting, http://step.esa.int/main/snap-supported-plugins/sen2cor, (accessed on 25 November 2021)) and the Sentinel Application Platform (SNAP, a common software architecture on which a collection of free open-source toolboxes for the scientific exploitation of Earth Observation missions, https://step.esa.int/main/download/snap-download, (accessed on 25 November 2021)). In this study, 28 Sentinel satellite images (https://scihub.copernicus.eu/dhus/#/home, (accessed on 24 November 2021)) were selected. Sen2Cor was used to perform radiometric calibration and atmospheric correction, and SNAP was used to resample each band of images. Table 2 presents the dates of selected Sentinel images.

### 2.3. Methods

#### 2.3.1. Empirical Regression Modelling for Retrieval of WQPs

In this study, three WQPs, including the Chl-a concentration, algal density, and turbidity, were used as retrieval objects, and different waveband ratio algorithms were used for retrieval. Band ratio algorithms are empirical algorithms based on the statistical relationship between a color index (i.e., band ratio) and a WQP [35]. Currently, there are relatively mature or more general retrieval algorithms, such as single bands [36], linear band combinations [30], band ratios [30], mixed-band combinations, such as Kab1 [37], the Green Difference Vegetation Index (GDVI) [38], 3BDA-like (Kivu) [39], the Vegetation Atmospheric Resistance Index (VARI) [40], etc. In this study, based on the selection of the above WQPs retrieval algorithms, a number of related parameters between the regression data and the measured water quality data were calculated (see Section 2.3.2 for specific parameters and equations) to screen out the best WQP algorithms in the study area. This part uses MS Office Excel to input the band combination formula, and the generated result is used as the independent variable of the retrieval regression equation. The band algorithm is presented in Table 3, and *i*, *j,* and *k* refer to different remote sensing bands.

The empirical model used in the regression of in situ sampling and band combinations consisted of the following model equations [9], where *R_rs_*(*λ*) is the reflectance corresponding to the Landsat 8 and Sentinel-2 bands, and *a*, *b*, and *c* are regression model constants.

(2) $$\mathrm{Linear}\;a\times R_{rs}\left(\lambda \right)+b$$

(3) $$\mathrm{Polynomial}\;a\times R_{rs}\left(\lambda \right)+b\times R_{rs}\left(\lambda \right)+c\;$$

(4) $$\mathrm{Logarithmic}\;a\times log_{10}R_{rs}\left(\lambda \right)+b\;$$

(5) $$\mathrm{Power}\;a\times R_{rs\left(\lambda \right)}^{b}\;$$

(6) $$\mathrm{Exponential}\;a\times e^{b*R_{rs}(\lambda )}\;$$

In this paper, training datasets and validation datasets were selected according to the ratio of 7:3. The data of 10 sampling points were used in the regression modeling of Landsat OLI data, the remaining 4 data points were used for model validation, 19 sampling points were used in the regression modeling of Sentinel MSI data, and the remaining 9 data points were used for model validation. The Statistical Product and Service Solutions (SPSS) software was selected to take the result obtained by the band combination as the independent variable, and the WQPs values of the regression points were used as the dependent variable to generate the water quality retrieval formula. Afterwards, the band combination results corresponding to the validation datasets were brought back into the retrieval formula to generate the statistical indicators mentioned in Section 2.3.2.

#### 2.3.2. WQPs Retrieval Performance Analysis Metrics

**Figure 2:**
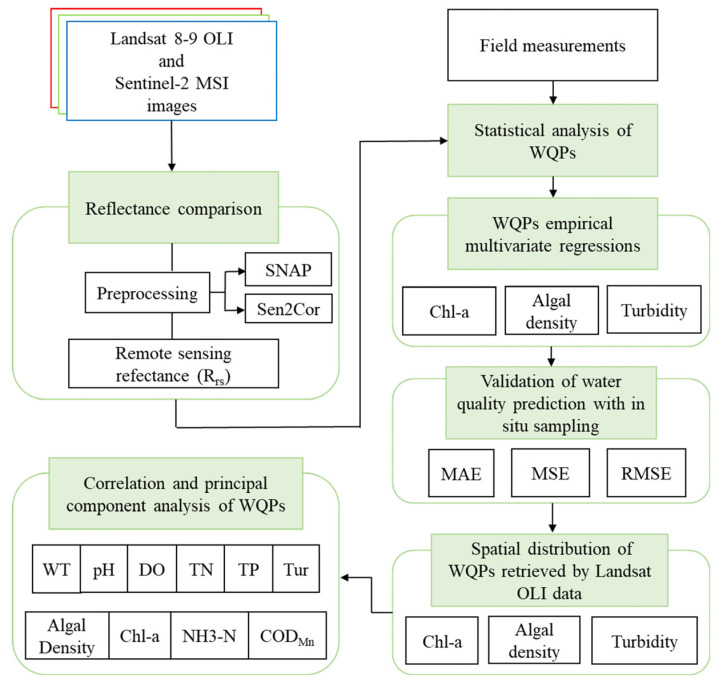
Analytical framework applied in the present study.

To determine and compare the performance of the empirical model in retrieving various WQPs, we compared the retrieval results with the measured water quality using the following formulas: Correlation coefficient (*r*), coefficient of determination (*R*^2^), standard deviation (SD), standard error (SE), coefficient of variation (CV), mean absolute error (MAE), mean square error (MSE), and root mean square error (RMSE). Table 4 lists the calculation formula for each index. The flowchart in Figure 2 summarizes the overall flow of the research.

## 3. Results

### 3.1. Behavior Parameters of In Situ WQPs at Sampling Station

Table 5 shows the average Chl-a concentration, turbidity, algal density, and WT values obtained from the monitoring activities from November 2020 to February 2022. After removing some null values or outliers with obvious order-of-magnitude error, the data showed that the Chl-a concentration change in Shanmei Reservoir fluctuated between 5.24 ± 3.04 μg/L and the maximum value of 23.11 μg/L. The algal density fluctuated the most, with an average value of 4.46 × 10^6^ cells/L, and a CV value of 141.17%. The turbidity fluctuated between 3.97 ± 2.84 (NTU) and its CV value reached 71.50%. The WT fluctuation was the smallest, with a CV value of only 20.70%, between 16.26 and 34.64 °C. In 2020–2022, the nutrient status and productivity of the reservoirs in the study area were relatively low.

### 3.2. Landsat 8-9 OLI and Sentinel-2 MSI Reflectance (R_rs_(λ)) Comparison

**Figure 3:**
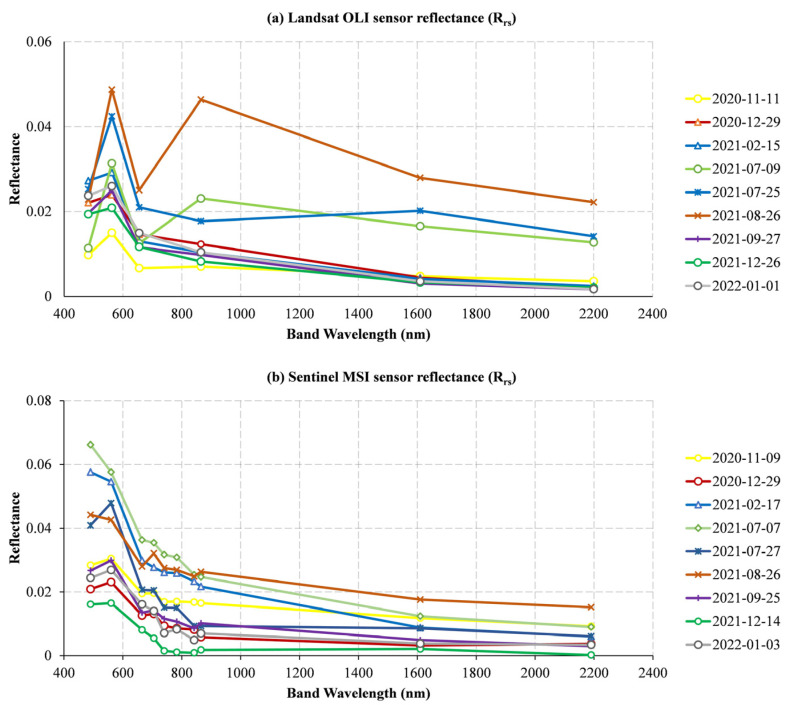
Reflectance variation in different bands for (**a**) Landsat OLI data and (**b**) Sentinel MSI data.

The remote sensing reflectance of Landsat OLI and Sentinel MSI at the same or similar time points were compared, as shown in Figure 3. The reflectivity of Landsat OLI sensor band ranged from 0 to 0.05, and that of the Sentinel MSI sensor band ranged from 0 to 0.07. In general, with the increase in wavelength, the trend of the increase/decrease in the reflectance value of the two sensors at the water quality monitoring point is similar. Among them, the reflectivity of the visible light band (450–680 nm) and NIR band (785–900 nm) was higher than that of the SWIR band (1560–2300 nm), and the green (B3) band (525–600 nm) has the highest reflectivity in the visible band (the conclusion does not include abnormal dates, such as 17 February 2021, 7 July 2021, and 26 August 2021—in these three dates, the reflectivity of the blue (B2) band (450–515 nm) of Sentinel MSI data is higher than that of the green (B3) band (525–600 nm), while the reflectivity of some visible bands of Landsat OLI data is lower than that of the SWIR band (1560–2300 nm)).

### 3.3. WQPs Regressions from Landsat OLI and Sentinel MSI Data

#### 3.3.1. Regression of Chl-a Concentration

Table 6 and Table 7 show the band combinations and their regression models with a good fitting effect for the two sensors. By comparing the Chl-a concentration estimated by Sentinel MSI data and Landsat OLI data with the in situ Chl-a concentration, both showed higher *R*^2^ values. This confirmed the plausibility of the developed regression model for estimating the Chl-a concentration in the case study reservoir. For Landsat OLI data, we found the three best regression equations fits for Chl-a concentration retrieval using a combination of coastal aerosol (B1), blue (B2), green (B3), and NIR (B4) bands, and the highest value of *R*^2^ is 0.70. Compared with the Sentinel MSI regression equations using the red (B4), Vegetation red edge1 (B5), and Vegetation red edge2 (B6) bands to predict Chl-a concentrations, the accuracy was improved by a minimum of 12.90%.

#### 3.3.2. Regression of Algal Density

Table 8 and Table 9 summarize the specific situation of the satellite sensors in the retrieval of algal density in the reservoir. The Landsat OLI data obtained the three best results, and the Sentinel-2 satellite obtained the two best results. The three results of Landsat OLI data include coastal aerosol (B1), blue (B2), green (B3), and red(B4) bands, and Sentinel MSI data include the red (B4), Vegetation red edge1 (B5), and Vegetation red edge2 (B6) bands. The best result for Landsat OLI algal density estimation was obtained using the univariate linear regression equation of coastal aerosol (B1), green (B3), and red(B4) bands with *R*^2^ of 0.82, and for Sentinel MSI data using the red (B4), Vegetation red edge1 (B5), and Vegetation red edge2 (B6) bands, where the *R*^2^ of the best regression equation is 0.61. It can be seen that the accuracy of the regression formula of Landsat OLI data on algal density was at least 26.23% higher than that of Sentinel MSI data. The results show that although the measured algal density values have a wide range, the Landsat OLI retrieval regression equation can verify the validity of the established model and predict algal density to a certain extent.

#### 3.3.3. Regression of Turbidity

Table 10 and Table 11 summarize the best regression models for turbidity retrieval. For the Landsat OLI sensor, the coastal aerosol (B1), blue (B2), green (B3), and red(B4) bands were dominant in the retrieval of turbidity in the reservoir. For the Sentinel-2 MSI sensor, the coastal aerosol (B1), blue (B2), and green (B3) bands were dominant in the retrieval of turbidity in the reservoir. The univariate linear regression model of the Landsat OLI data performed the best in retrieving reservoir turbidity with significantly higher accuracy than the Sentinel MSI data. On the other hand, Sentinel MSI data have poor regression accuracy and are almost unusable. Specifically, the best estimate of turbidity using Landsat OLI data was *R*^2^ = 0.71, while the best estimate of turbidity by Sentinel MSI data was only 0.14. This demonstrates the decisive advantage of the Landsat OLI data in the retrieval of reservoir turbidity in the study area.

### 3.4. Validation of Water Quality Prediction with In Situ Sampling

The regression model developed in Section 3.3 is verified with the remaining measured data, and the verification results are listed in Table 12, including the in situ statistics, which were used in the model calibration. It can be seen from the table that Landsat OLI data tend to overestimate the values of chlorophyll-a and algal density, the Sentinel MSI data-based model tends to underestimate the values of chlorophyll-a and algal density, while the values of turbidity are both underestimated. From the three indicators of SD, SE, and CV, the accuracy of WQPs simulated by Landsat OLI data is more accurate than Sentinel MSI data.

To assess the merits and compare different WQP regression equations, all the measured data were entered into the regression formula, and the optimal results of the two satellite regression parameters are shown in Table 13. The regression model developed by Landsat OLI data has obvious advantages, and the three types of regression parameters including MAE, MSE, and RMSE were all smaller than the regression model based on Sentinel-2, which indicated that Landsat OLI data and its regression model were applicable to this study area.

**Figure 4:**
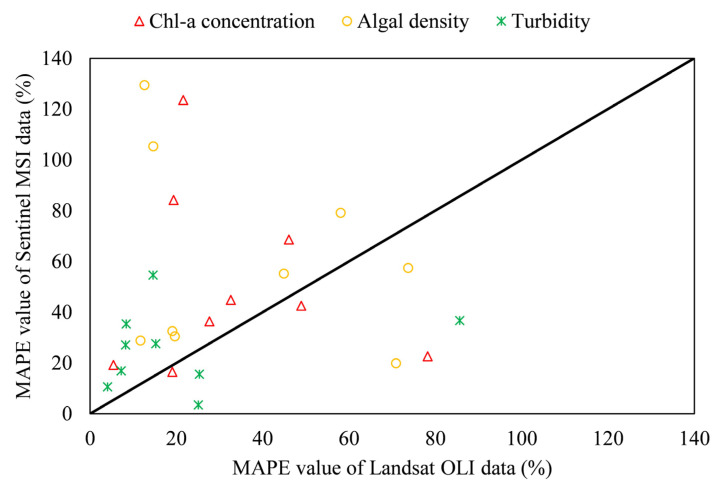
MAPE value of Landsat OLI and Sentinel MSI data with the same or similar time points.

According to Section 3.2, the Landsat OLI data and Sentinel MSI images have nine images with the same or similar time nodes. Therefore, the simulated water quality results of the regression formula for the nine time periods were compared with the measured data, as shown in Figure 4. Compared with the simulation results of the Sentinel MSI data regression model, the simulation results of Landsat OLI data agreed more with the measured water quality data, which can also explain the applicability of Landsat OLI data in the study area.

**Figure 5:**
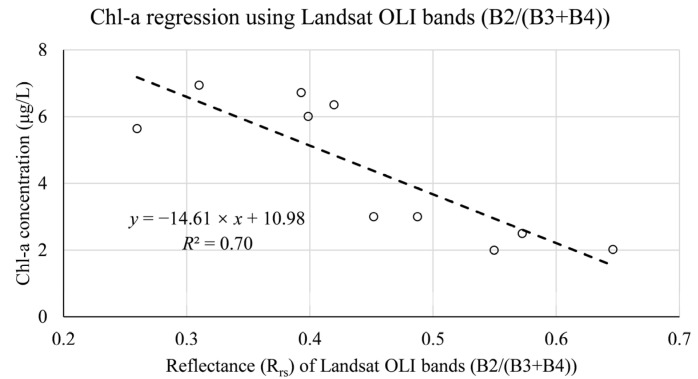
In situ-derived Chl-a concentration and Landsat OLI bands reflectance from training datasets.

**Figure 6:**
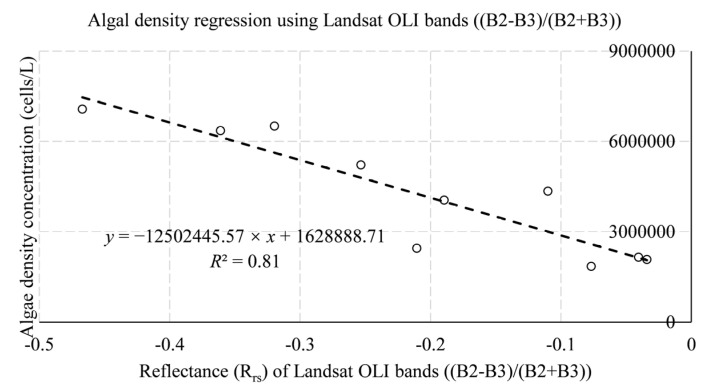
In situ-derived algal density and Landsat OLI bands reflectance from training datasets.

**Figure 7:**
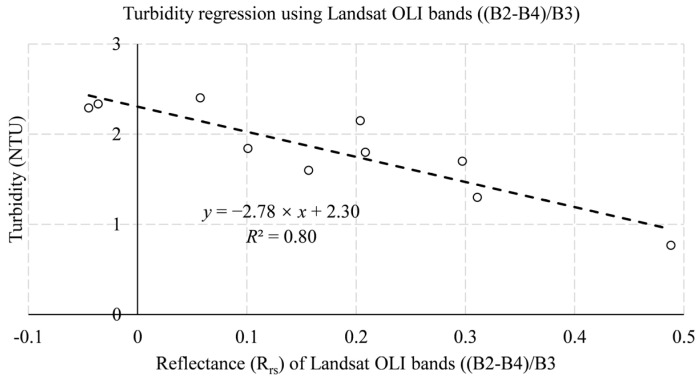
In situ-derived turbidity and Landsat OLI bands reflectance from training datasets.

In conclusion, the Landsat OLI data are determined to be the retrieval data for this study. Figure 5, Figure 6 and Figure 7 show the best regression models for estimating chlorophyll a concentration, algal density, and turbidity from Landsat OLI data.

### 3.5. Spatial Distribution of WQPs Retrieved by Landsat OLI Data

#### 3.5.1. Distribution of Chl-a Concentration

**Figure 8:**
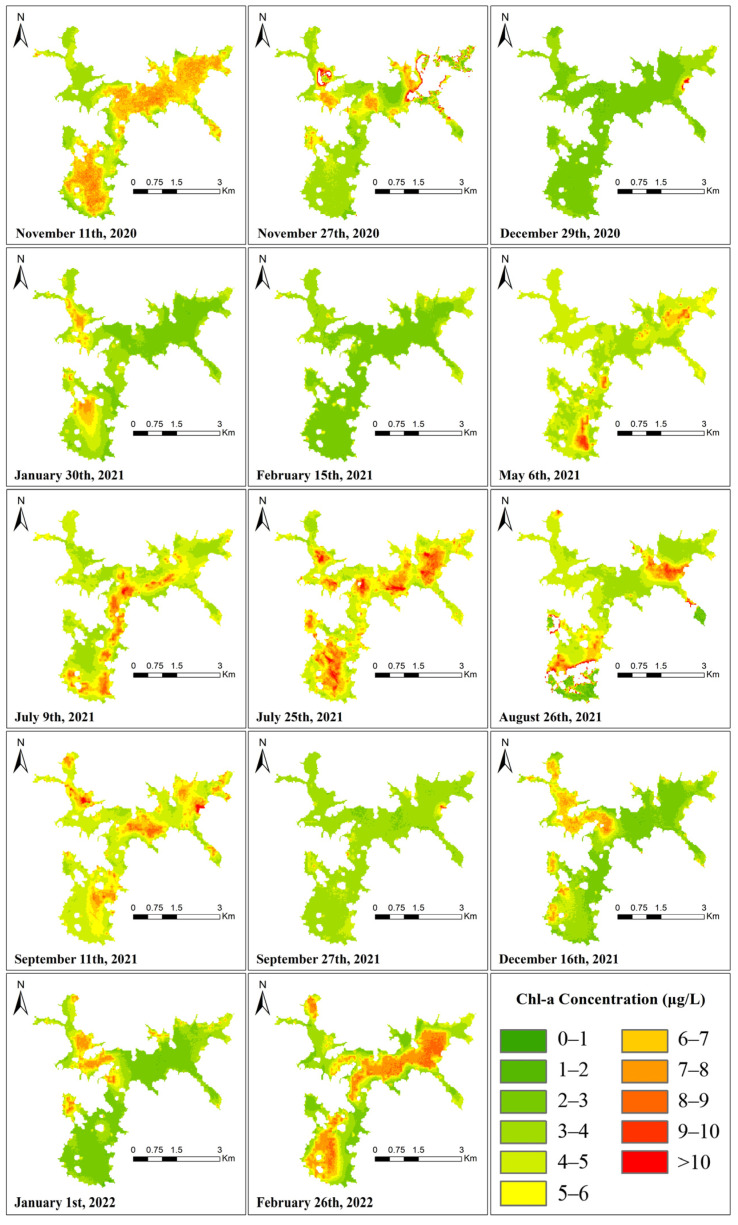
Spatial distribution of Chl-a concentration from Landsat OLI data.

Using the Chl-a concentration regression equation generated in Section 3.3 to retrieve the Landsat 8-9 image, the spatial distribution of Chl-a in the Shanmei Reservoir at each time node is shown in Figure 8. The presence of clouds over the study area resulted in abnormal Chl-a concentrations (such as the upper-right area of the 27 November 2020 image and the lower-left area of the 26 August 2021 image, which have been removed from the image). Although the classified color changes of the Chl-a concentration from 2020 to 2022 in the figure were evident, the concentration range was 0.20–28.36 μg/L, the overall Chl-a concentration was low, and the average concentration was 4.25 μg/L. Even though the retrieval results are affected by the limitations of the regression equation and the influence of cloudiness, the distribution and changes in the Chl-a concentration can still be clearly observed in the figure.

#### 3.5.2. Distribution of Algal Density

**Figure 9:**
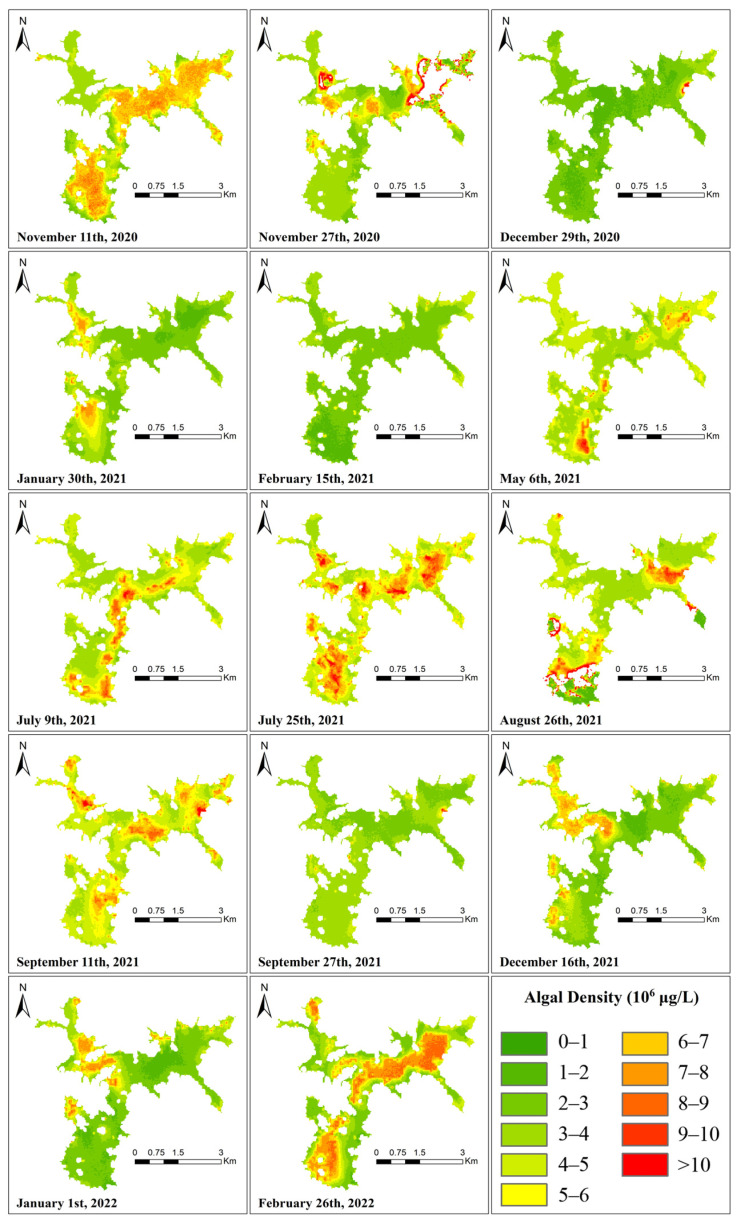
Spatial distribution of algal density concentration from Landsat OLI data.

Figure 9 shows the results of the retrieval using the algal density regression equation. The density range of algal density in Shanmei Reservoir was between 5.77 × 10^4^ and 3.02 × 10^7^ cells/L, and the spatial distribution was relatively consistent with the Chl-a concentration. Although the color change in the algal density classification was stronger overall than that of the Chl-a concentration, the algal density had more low values and fewer high values, so the actual numerical change was not high. The average value from 2020 to 2022 was 4.11 × 10^6^ cells/L.

#### 3.5.3. Distribution of Turbidity

**Figure 10:**
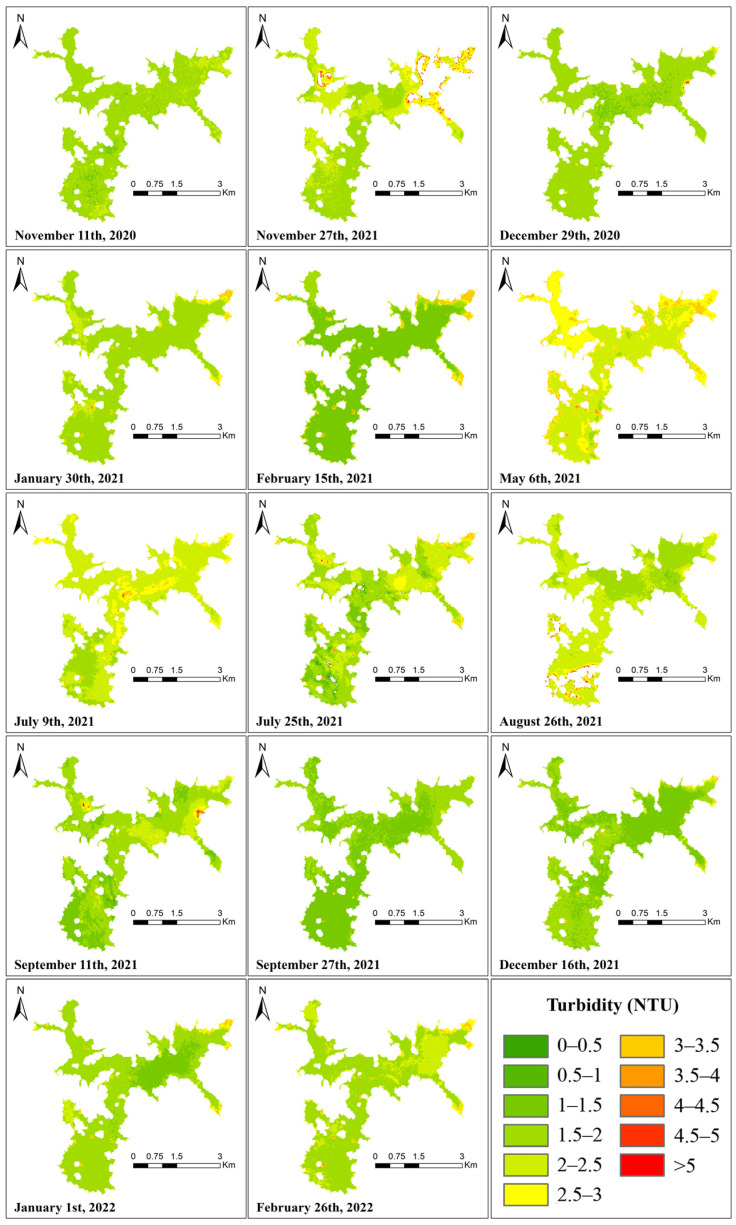
Spatial distribution of turbidity from Landsat OLI data.

Figure 10 shows the results of the retrieval using the turbidity regression equation. The turbidity variation ranged from 0.25–9.23 NTU. The figure shows that, compared with the Chl-a concentration and algal density distribution maps, the turbidity variation of Shanmei Reservoir was small. Except for the influence of the cloud layer on 27 November 2020 and 26 August 2021, only on 6 May 2021 and 9 July 2021,was there more significant variation, and the average turbidity reached 2.50 and 2.26 NTU. The average turbidity during the study period was only 1.86 NTU.

### 3.6. Relationship between Reservoir WPQs

**Figure 11:**
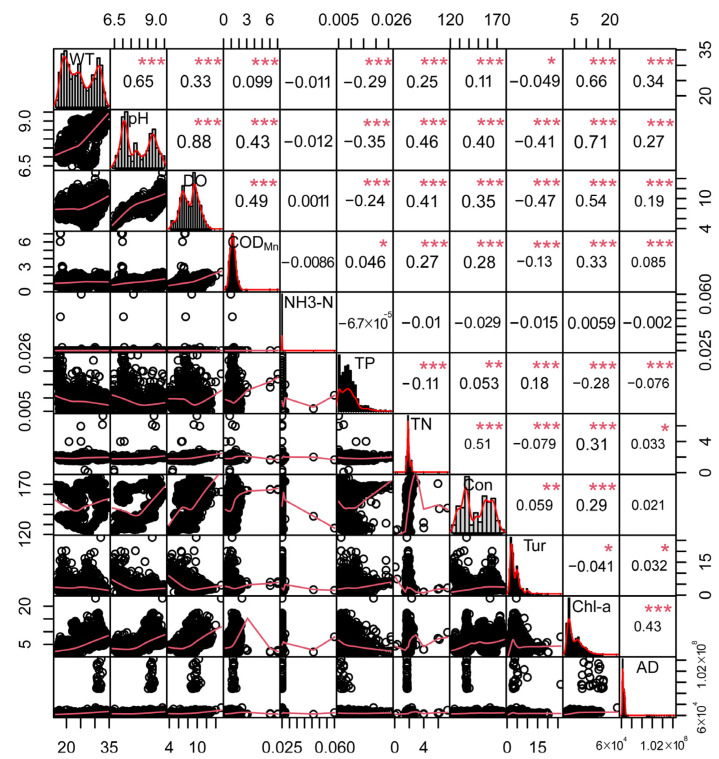
Correlation coefficient diagram between WQPs. Con, conductivity, Tur, turbidity, AD, algal density. The diagonal line gives the distribution, histogram, and density curve of WQPs. The lower triangle (the lower-left corner of the diagonal) gives the scatter diagram and the upper triangle (the upper-right corner of the diagonal) between the two WQPs. The value represents the correlation coefficient of the two variables. The larger the value, the greater the correlation degree; the asterisk indicates the degree of significance, * indicates *p* < 0.05, ** indicates *p* < 0.01, *** indicates *p* < 0.001.

In Figure 8 and Figure 9, the inversion results of the algae density and Chl-a concentration are very similar, indicating that there is a certain degree of relationship between the algal density and Chl-a concentration. However, when the Chl-a concentration and algae density have local high values, there is no obvious numerical and spatial change in turbidity. In order to better judge its relationship with water quality, correlation analysis was performed on all obtained WQPs using SPSS, as shown in Figure 11.

We can see that for the three WQPs of the Chl-a concentration, algal density, and turbidity involved in this study (located in the 3 × 3 square in the lower right corner of Figure 11), Chl-a concentration is correlated with algal density at the significance level of 0.001, and the correlation coefficient is 0.43, while the correlation coefficient of turbidity with Chl-a and algal density is almost 0, which is more consistent with the results shown in the retrieval image of the study area.

In other aspects of water quality (the following correlation coefficients are related at the significance level of 0.001), the average correlation coefficient between WT and pH reaches 0.65. The correlation coefficients between Chl-a and WT, DO, and pH are 0.66, 0.71, and 0.54, respectively, the correlation coefficient between TN and conductivity is 0.51, and the correlation between pH and DO is the highest, reaching 0.88. It can be seen that WT, pH, DO, TN, conductivity, and Chl-a concentration are important factors affecting the water quality of the reservoir.

**Figure 12:**
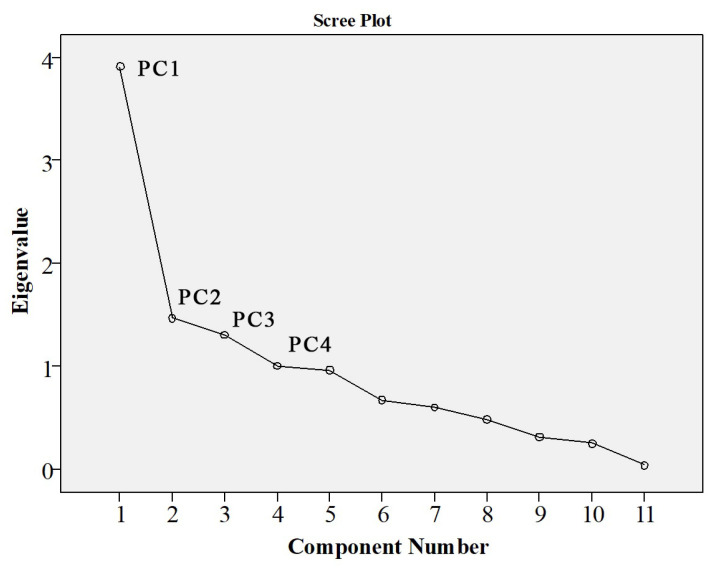
The Scree plot of WQPs.

The analysis of nine types of water quality factors using principal component analysis (PCA) in SPSS is shown in Figure 12. It can be seen that four principal components explaining 69.88% of the total variation were sufficient for the study. PC1 accounted for 35.57%, PC2 explained 13.34%, PC3 accounted for 11.87%, and PC4 explained 9.10% of the total variation, as calculated by loadings for a cumulative percentage of variance using SPSS.

## 4. Discussion

### 4.1. Analysis of Current Water Quality Status and Relationship between WQPs

It can be seen that there is no obvious seasonal or monthly variation law of the Chl-a concentration, algal density, and turbidity in Shanmei Reservoir. The average annual concentration of Chl-a in the reservoir is 4.25 μg/L. The numerical variation trend of algal density is similar to that of Chl-a concentration, and it is also maintained at a low level. For turbidity, its value and spatial distribution have been kept in a stable state, and the multi-year average is only 1.86 NTU. The retrieval results of the three WQPs also show that the eutrophication level in the study area is low.

On the other hand, as can be seen from the reflectivity of each band in Figure 3, the reflectivity of the green (B3) band is higher than those of the longer wavelength bands, partly because of atmospheric processes and partly because of the presence of phytoplankton [35], which explains the peak of the green (B3) band in Figure 3. In the red-edge (705–782 nm) bands, the lack of phytoplankton makes the peak near the 705 nm wavelength in eutrophic lakes visible, and the peak of reflectance is more obvious. In oligotrophic lakes, the reflectance is close to 0 [41], which is in line with the characteristics of the oligotrophic state of the Shanmei Reservoir.

However, the spatial distribution of the Chl-a concentration and algal density (Figure 8 and Figure 9) showed that there are high local values of Chl-a concentration and algal density at the remaining time points, except 29 December 2020, 15 February 2021, and 27 September 2021. On 11 November 2020, 25 July 2021, 11 September 2021, and 26 February 2022, the values of cha-a and algal density in the whole reservoir are high, indicating that there is still a risk of eutrophication in the reservoir.

**Figure 13:**
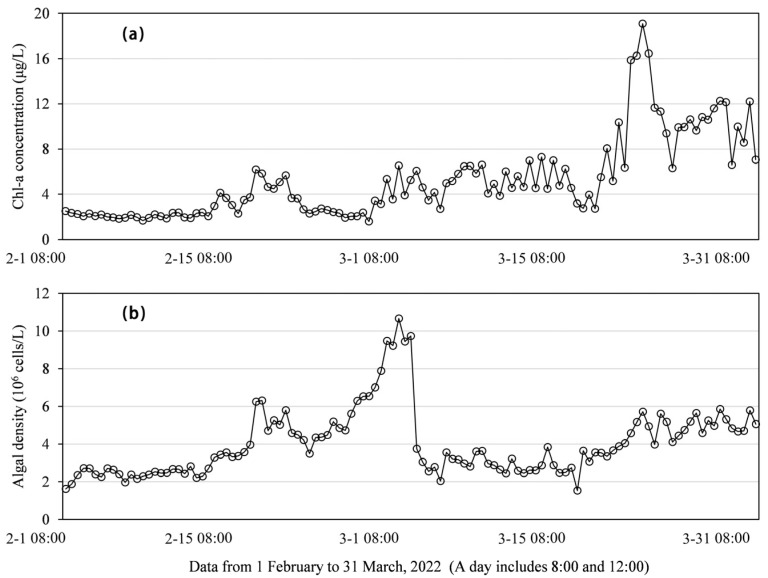
Daily values of (**a**) Chl-a concentration and (**b**) algal density.

It is worth noting that the study area in February is in winter, and the algae density and chlorophyll concentration should normally be at low values, but there are obvious differences between the retrieval images in February 2022 and February 2021. On 15 February 2021, the Chl-a concentration and algae density values were relatively low, and on 26 February 2022, the Chl-a concentration and algae density in the reservoir showed large-area high values. In situ data (Figure 13) show that the average Chl-a concentration in February 2021 is 2.15, the value in March 2021 is 3.96, and the value in February 2022 is 3.07, but the value in March 2022 increased to 8.11. This shows that starting from the end of February 2022, the eutrophic level of the reservoir begins to increase, and control measures and continuous observation are urgently needed. This also confirms the feasibility and accuracy of remote sensing inversion in reservoir water quality monitoring.

It can also be seen that the Chl-a concentration on 9 July 2021 has only a small number of high values in the middle and south of the reservoir, and the high values have been distributed to the whole region on 25 July, approximately half a month later. The Chl-a concentration and algal density showed the characteristics of a fast diffusion speed and long duration.

Table 14 shows that pH, DO, Chl-a concentration, WT, TN, and COD_Mn_ dominated PC1, which explained 35.57% of the total variance, and conductivity, algal density, and WT dominated PC2, which accounted for 14.91%, indicating the importance of pH, DO, Chl-a concentration, WT, TN, COD_Mn_, and conductivity in estimating water quality in the study area.

At present, the retrieval of the COD_Mn_ mainly uses conventional satellite remote sensing (such as GF series satellites, Landsat series satellites) [42,43], while the retrieval of DO and TN using hyperspectral remote sensing has higher accuracy [44,45,46]. Combined with the results of the above-related studies, it can be considered that the water quality of Shanmei Reservoir can be better evaluated by measuring pH, conductivity, and WT at the monitoring station, or by establishing the regression fitting equations between Chl-a, algae density, and turbidity and DO, COD_Mn_, and TN.

### 4.2. Selection and Applicability Analysis of Retrieval Band and Algorithm

Taimi et al. used the Olushandja dam in Namibia as a case study and developed a retrieval algorithm based on regression analysis using Landsat-8 reflection value and water quality data such as turbidity, TSS, ammonia, TN, TP, and total algae measured on-site. They found that a regression analysis using blue (B2), green (B3), red (B4), and NIR (B5) bands yields good results [47]. Willibroad et al. compared four established satellite reflectance algorithms to estimate the Chl-a concentration of Lake Chad, and the results showed that the 3BDA algorithm composed of blue (B2), green (B3), and NIR (B4) bands of Landsat-8 has higher accuracy [48]. Yashon et al. used Sentinel-2 and Landsat-8 data products to evaluate and retrieve Chl-a concentration, suspended particulate matter, and turbidity, and the results showed that Landsat-8 data performed better in retrieving WQPs, and they found that, in blue waters, owing to the high reflectivity of green algae, green and blue bands are suitable for the detection of algal blooms [17]. In this study, in the regression formula generated using Landsat OLI reflectance and water quality data, the regression formula for Chl-a concentration is associated with blue (B2), green (B3), and red (B4) bands, the algal density regression formula is highly correlated with blue (B2) and green (B3) bands, and the turbidity regression formula is correlated with blue (B2), green (B3), and red (B4) bands, which is consistent with the above conclusion. However, Figure 4 shows that Landsat OLI data and Sentinel MSI data use the same band combinations, but MAPE values show obvious differences, indicating that the band combinations will show different simulation effects on various remote sensing data, which may also be the reason for the large difference in the accuracy between the two remote sensing data simulation WQPs regression.

The optical properties of inland waters are very different between water bodies, and there are also significant differences within water bodies. These problems hinder the development of inland water algorithms and typically limit their applicability to different water bodies [5]. Lai et al. retrieved the concentration and distribution of Chl-a in the Guanting Reservoir based on the measured data in different years and Landsat-8 images and 22 algorithm formulas, including SABI, KIVU, Apple, and other vegetation indicators, and found that there was a strong correlation between the pixel values of adjacent reservoirs in the same image, so the Chl-a estimation model can be applied to each other [49]. Richard et al. evaluated the performance of 29 algorithms that used satellite spectral data to retrieve Chl-a concentration in two temperate inland lakes, to use it as an indicator of the general state of algal density and potential algal density. Although the two lakes differ in background water quality, size, and shape, the results support multiple sensors utilizing a specific set of algorithms to detect potential algal blooms by using Chl-a concentration as a proxy [50]. Therefore, whether the WQPs regression formula generated in this study can be applied to other reservoirs (near the study area or with some of the same characteristics as the study area) is a future research direction.

### 4.3. WQPs Regression Formula and Retrieval Error Analysis

The preprocessing process of Landsat OLI data and Sentinel MSI data is very important, and different processing methods will affect the conversion from top of atmosphere (TOA) reflectance to surface reflectance, thus affecting the regression results of WQPs. In this study, the method of downloading and processing data selected the most mainstream method in the current research, so it can ensure the accuracy of the research results to the greatest extent, even if there are still some inevitable errors.

Another instance of error originates from the adjacency effect of the adjacent land pixels, which is known as the border effect. Inland water bodies are surrounded mostly by land, and border effects are especially significant in areas with raised, undulating topography around the water body [51]. This means that light from objects around the body of the water can change the radiance reaching the sensor, and large parts of the sky may also be blocked by the ground surface (e.g., vegetation) [5], making it impossible to obtain the true WQPs at the water boundary accurately.

In addition, the date of collection of water quality data can also be a source of error when comparing it with remote sensing data products from different sensors. Because the revisit period of Landsat 8-9 satellite combination is 8 days, and the combination of Sentinel-2A/2B satellites is 5 days, it is difficult to ensure that the data time of the two satellites is completely consistent, which affects the comparative analysis of the WQPs regression equation. Therefore, in addition to achieving good performance in the preprocessing and data regression fitting stages, it is important to ensure that the data collection dates are closer to each other. In this study, WQPs data were obtained daily, but the time difference of remote sensing images caused uncertainty in the fitting of WQPs. Therefore, future research could create a new and more reliable method to quantify changes in WQPs with a higher temporal resolution by combining products from different remote sensing data sources, together with appropriate water quality estimation algorithms. Simultaneously, the different performances of the understanding algorithm and remote sensing image pairs should also be considered. For example, Yashon et al. adjusted the two remote sensing datasets by band adjustment, performed preprocessing such as atmospheric correction and normalized reflectance and then used the standardized data to retrieve reservoir WQPs [17].

The validity and accuracy of elemental determinations of water quality depend on the satellite sensors used, the methods employed, and the nature of the waters studied. In this study, regression results for Chl-a concentration, algal density, and turbidity demonstrated the potential of optical satellite remote sensing reflectance data for cost-effective, large-scale, and high-frequency use in monitoring optically active water elements. The purpose of remote sensing retrieval of water quality is to provide real-time assessment of current and future water quality monitoring to prevent water quality deterioration. Despite the good water quality of the reservoirs presented in this study, we recommend their continuous monitoring and management through regression simulation and the retrieval of other important WQPs, such as DO, COD_Mn_, and TN, so as to ensure the good water quality of the reservoir.

### 4.4. Research Limitations and Prospects

Based on the WQPs regression algorithm obtained from a single monitoring point, this study determines the key water quality characteristics of the reservoir and provides a more feasible idea for inland waters with few monitoring points. However, due to the limitation of the number of water quality monitoring stations, the problem of a small amount of matching data is inevitable. The predicted value of water quality obtained by simulation cannot be aptly compared with the actual value of water quality. The actual spatial and numerical changes in water quality are difficult to quantify, and the regression model will also be affected by the amount of data. With the extension of monitoring time, the regression coefficient may change, but when the data reaches a certain amount, a more accurate and stable regression equation can often be obtained. At the same time, the alternation of day and night, temperature, the intensity of human activities, and the action of aquatic organisms will indeed directly or indirectly affect water quality [52,53,54]. However, the transit time of the remote sensing satellites used in this study is in the morning, so the change in water quality at night is not considered. In addition, Sun et al. believe that non-optically active parameters may be highly correlated with optically active substances, such as Chl-a, TSM, and CDOM [55], so TN, TP, and COD can be estimated remotely [15]. At present, some scholars have developed several statistical techniques with empirical and machine learning algorithms to determine the relationship between reflectance and non-optically active parameters in inland waters with the help of hyperspectral images [46,56]. As mentioned in Section 4.1, the three WQPs in this study have a certain correlation with some non-optically active water quality parameters (such as DO, COD_Mn_, and TN). Therefore, the WQPs of regional non-optically active water quality can be estimated through machine learning algorithms. In the ideal future, the acquisition frequency and accuracy of satellite images will be the same as that of water sample data, so as to reduce the time difference between different satellite images. At the same time, more matching data can be obtained by adding monitoring points or stations at different locations, as was performed by Curtarelli et al. who arranged them in the reservoir near the dam, in the middle of the reservoir, at the tail of the reservoir, and near the tributary [57]. In addition, the impact on the water quality of inland reservoirs can also be studied in terms of hydrological changes such as water volume and reservoir depth [58], so as to more comprehensively judge the current status and future trends of reservoir water quality. At the same time, for remote sensing data, preprocessing and adjacency affect the selection or development of corresponding algorithms for correction and the retrieval of more accurate water quality data, which can be used for water resources management and environmental protection planning.

## 5. Conclusions

This study compared the accuracy of Landsat 8-9 OLI and Sentinel 2 MSI sensors for the retrieval of Chl-a, algal density, and turbidity in the reservoir. Both types of satellite data showed high reflectivity in the green (B3) band. The results of the empirical multiple-regression model show that the *R*^2^ and validation parameters (MAE, MSE, and RMSE) of the Landsat OLI fitting equation are better than Sentinel MSI data. Therefore, Landsat OLI data have better application potential in this study area. The 2020–2022 reservoir water quality images retrieved from Landsat OLI data show that the multi-month average values of reservoir WQPs are low. However, from the end of February 2022, the Chl-a concentration and algal density in the reservoir gradually increased, and local high values appeared. Therefore, continuous attention and corresponding water quality management measures are still needed. The results of correlation analysis and principal component analysis show that the water quality of Shanmei Reservoir can be evaluated more accurately and quickly by measuring the pH, conductivity, and WT of the monitoring station, or by establishing the regression fitting equation between Chl-a, algae density, and turbidity and DO, COD_Mn_, and TN. In the future, to improve the accuracy of the estimation of the overall water quality status of the reservoir, new methods can be developed to monitor, fit, and retrieve more factors that can represent the water quality status, or understand the impact of the algorithm on the different performances of remote sensing images to conduct frequent water quality assessments. Simultaneously, we can also apply the regression equation from the study area to verify the accuracy of the regression formula in adjacent waters or similar waters.

## Figures and Tables

**Table 1:** Dates of selected Landsat 8-9 images.

Number	Year	Image Date	Number	Year	Image Date
1	2020	11 November	8	2021	25 July
2	2020	27 November	9	2021	26 August
3	2020	29 December	10	2021	11 September
4	2021	30 January	11	2021	27 September
5	2021	15 February	12	2021	16 December
6	2021	6 May	13	2022	1 January
7	2021	9 July	14	2022	26 February

**Table 2:** Dates of selected Sentinel images.

Number	Year	Image Date	Number	Year	Image Date
1	2020	9 November	15	2021	16 August
2	2021	29 December	16	2021	26 August
3	2021	13 January	17	2021	5 September
4	2021	18 January	18	2021	25 September
5	2021	23 January	19	2021	5 October
6	2021	2 February	20	2021	14 November
7	2021	17 February	21	2021	24 November
8	2021	9 March	22	2021	04 December
9	2021	24 March	23	2021	09 December
10	2021	12 June	24	2021	14 December
11	2021	17 June	25	2021	19 December
12	2021	7 July	26	2021	29 December
13	2021	22 July	27	2022	3 January
14	2021	27 July	28	2022	8 January

**Table 3:** The algorithms used for calculation of WQPs.

Band Combinations	Landsat OLI Data	Sentinel MSI Data
Single bands	B_L8*i*_	B_S2*i*_
Linear band combination	B_L8*i*_ + B_L8*j*_	B_S2*i*_ + B_S2*j*_
Band ratios	B_L8*i*_/B_L8*j*_	B_S2*i*_/B_S2*j*_
Mixed band combinations	(B_L8*i*_ + B_L8*j*_)/B_L8*k*_	(B_S2*i*_ + B_S2*j*_)/B_S2*k*_

**Table 4:** WQPs statistical indicators.

Name	Equation
Correlation coefficient	$r=\frac{\sum_{i=1}^{n}\left(\left(X_{i}^{Measured}-{\bar{X}}^{Measured}\right)\left(Y_{i}^{Estimated}-{\bar{Y}}^{Estimated}\right)\right)}{\sqrt{\sum_{i=1}^{n}{\left(X_{i}^{Measured}-{\bar{X}}^{Measured}\right)}^{2}}\sqrt{\sum_{i=1}^{n}(Y_{i}^{Estimated}-{\bar{Y}}^{Estimated}{)}^{2}}}$
Determination coefficient	$R^{2}=1-\frac{\sum_{i=1}^{n}\left(X_{i}^{Measured}-X_{i}^{E\mathrm{stimated}}\right)}{\sum_{i=1}^{n}\left(X_{i}^{Measured}-{\bar{X}}_{}^{E\mathrm{stimated}}\right)}$
Standard deviation (SD)	$\mathrm{SD}=\sqrt{\frac{1}{n}\times \sum_{i}^{n}{\left(X_{i}-\bar{X}\right)}^{2}}$
Standard error (SE)	$\mathrm{SE}=\frac{\mathrm{SD}}{\sqrt{n}}$
Coefficient of variation (CV)	$\mathrm{CV}=\frac{\mathrm{SD}}{\bar{X}}$
Mean absolute error (MAE)	$\mathrm{MAE}=\frac{1}{n}\times \sum_{i=1}^{n}\left|X_{i}^{Estimated}-X_{i}^{Measured}\right|$
Mean square error (MSE)	$\mathrm{MSE}=\frac{1}{n}\times {\sum_{i=1}^{n}\left(X_{i}^{Estimated}-X_{i}^{Measured}\right)}^{2}$
Root mean square error (RMSE)	$\mathrm{RMSE}=\sqrt{\mathrm{MSE}}$
Mean absolute percentage error (MAPE)	$\mathrm{MAPE}=\frac{100}{n}\times \sum_{i=1}^{n}\left(\frac{X_{i}^{Estimated}-X_{i}^{Measured}}{X_{i}^{Estimated}}\right)$

**Table 5:** Descriptive statistics from Shanmei Reservoir.

Parameters	Statistical Indicators	Numerical Value
Chl-a concentration	min-max (μg/L)	1.00–23.11
	Average ± σ ^1^ (μg/L)	5.24 ± 3.04
	CV(%)	58.12
	n ^1^	2819
Algal Density	min-max (10^6^ cells/L)	0.06–102.11
	Average ± σ (10^6^ cells/L)	4.46 ± 6.29
	CV (%)	141.17
	n	2819
Turbidity	min-max (NTU)	0.62–26.36
	Average ± σ (NTU)	3.97 ± 2.84
	CV(%)	71.50
	n	2819
WT	min-max (° C)	16.26–34.64
	Average ± σ (° C)	24.52 ± 5.08
	CV (%)	20.70
	n	2819

^1^ n, data number, σ, standard deviation.

**Table 6:** Regression model for the retrieval of Chl-a concentration using Landsat OLI training datasets.

No.	Landsat OLI Data Regression Model Equation for Chl-a Concentration Estimation	Band Combination (=*x*)	r	*R* ^2^
1	*y* = −14.61 × *x* + 10.98	B2/(B3 + B4)	−0.84	0.70
2	*y* = −11.50 × *x* + 2.50	(B2 − B3)/(B2 + B3)	−0.80	0.65
3	*y* = −10.40 × *x* + 7.93	B1/(B3 + B4)	−0.79	0.62

**Table 7:** Regression model for the retrieval of Chl-a concentration using Sentinel MSI training datasets.

No.	Sentinel MSI Data Regression Model Equation for Chl-a Concentration Estimation	Band Combination (=*x*)	r	*R* ^2^
1	*y* = 1183.26 × *x* + 2.62	B5 − (B4 + B6)/2	0.79	0.62
*2*	*y* = 2.57 × e^284.24×*x*^			0.59

**Table 8:** Regression model for the retrieval of algal density concentration using Landsat OLI training datasets.

No.	Landsat OLI Data Regression Model Equation for Algal Density Estimation	Band Combination (=*x*)	r	*R* ^2^
1	*y* = −19,789,532.32 × *x* + 8,686,780.32	B1/(B2 + B3 + B4)	−0.91	0.82
2	*y* = −12,502,445.57 × *x* + 1,628,888.71	(B2 − B3)/(B2 + B3)	−0.90	0.81
3	*y* = 3,292,817.47 × *x* − 1,084,109.44	(B1 − B3)/(B1 + B3)	0.88	0.77

**Table 9:** Regression model for the retrieval of algal density concentration using Sentinel MSI training datasets.

No.	Sentinel MSI Data Regression Model Equation for Algal Density Estimation	Band Combination (=*x*)	r	*R* ^2^
1	*y* = 1,227,118,587.08 × *x* + 2,142,593.11	B5 − (B4 + B6)/2	0.78	0.61
2	*y* = 1,981,923.63 × e^351.40×*x*^			0.53

**Table 10:** Regression model for the retrieval of turbidity concentration using Landsat OLI training datasets.

No.	Landsat OLI Data Regression Model Equation for Turbidity Estimation	Band Combination (=*x*)	r	*R* ^2^
1	*y* = −2.78 × *x* + 2.30	(B2 − B4)/B3	−0.84	0.80
2	*y* = −7.08 × *x* + 4.93	B2/(B1 + B3)	−0.78	0.71
3	*y* = −3.10 × *x* + 2.24	(B2 − B4)/(B2 + B4)	−0.73	0.61

**Table 11:** Regression model for the retrieval of turbidity concentration using Sentinel MSI training datasets.

No.	Sentinel MSI Data Regression Model Equation for Turbidity Estimation	Band Combination (=*x*)	r	*R* ^2^
1	*y* = −10.42 × *x* + 2.76	B1	−0.37	0.14
2	*y* = 3.44 × *x* + 0.46	B3/(B1 + B2)	0.35	0.12

**Table 12:** Descriptive statistics of the in situ and predicted WQPs values.

Water Quality Parameter	Data Source	No.	Min	Max	Average	SD	CV(%)	SE
Chl-a concentration (μg/L)	Landsat OLI	1	2.26	3.15	2.76	0.40	14.35	0.20
		2	2.48	3.45	2.91	0.40	13.74	0.20
		3	1.97	3.18	2.56	0.52	20.17	0.26
	In situ data for L-O validation	/	1.99	8.17	3.72	2.58	69.53	1.29
	Sentinel MSI	1	2.86	7.23	4.15	1.30	31.38	0.65
		2	2.72	7.79	3.92	1.51	38.59	0.76
	In situ data for S-M validation	/	1.93	3.91	2.64	0.60	22.75	0.30
Algae density	Landsat OLI	1	1.57 × 10^6^	2.80 × 10^6^	2.15 × 10^6^	5.21 × 10^5^	24.24	2.60 × 10^5^
		2	2.10 × 10^6^	3.15 × 10^6^	2.56 × 10^6^	4.35 × 10^5^	16.95	2.17 × 10^5^
		3	1.94 × 10^6^	3.03 × 10^6^	2.41 × 10^6^	4.50 × 10^5^	18.68	2.25 × 10^5^
	In situ data for L-O validation	/	1.80 × 10^6^	6.54 × 10^6^	4.08 × 10^6^	2.17 × 10^5^	53.29	1.09 × 10^5^
	Sentinel MSI	1	2.39 × 10^6^	6.93 × 10^6^	3.72 × 10^6^	1.35 × 10^5^	36.23	6.75 × 10^5^
		2	2.13 × 10^6^	7.80 × 10^6^	3.41 × 10^6^	1.70 × 10^6^	49.89	8.49 × 10^5^
	In situ data for S-M validation	/	1.59 × 10^6^	3.50 × 10^6^	2.54 × 10^6^	5.74 × 10^5^	22.56	2.87 × 10^5^
Turbidity (NTU)	Landsat OLI	1	1.28	1.52	1.39	0.09	6.12	0.04
		2	1.47	1.61	1.53	0.06	3.76	0.03
		3	1.44	1.60	1.51	0.06	3.99	0.03
	In situ data for L-O validation	/	2.34	6.44	4.69	1.52	32.37	0.76
	Sentinel MSI	1	2.32	2.60	2.51	0.08	3.19	0.04
		2	1.96	2.53	2.25	0.18	8.05	0.09
	In situ data for S-M validation	/	4.48	8.02	5.84	1.19	20.37	0.59

**Table 13:** Comparison of evaluation indexes of WQPs regression formula (brought into training and testing dataset together).

WQPs	Data Type	Band Combination (=*x*)	Regression Model Equation	MAE	MSE	RMSE
Chl-a concentration	Landsat OLI	B2/(B3 + B4)	*y* = −14.61 × *x* + 10.98	1.19	2.75	1.66
	Sentinel MSI	B5 − (B4 + B6)/2	*y* = 2.57 × e ^284.24×*x*^	1.12	2.92	1.71
Algal density	Landsat OLI	(B2 − B3)/(B2 + B3)	*y* = −12,502,445.57 × *x* + 1,628,888.71	9.19 × 10^5^	2.04 × 10^12^	1.43 × 10^6^
	Sentinel MSI	B5 − (B4 + B6)/2	*y* = 1,227,118,587.08 × *x* + 2,142,593.11	1.13 × 10^6^	2.77 × 10^12^	1.66 × 10^6^
Turbidity	Landsat OLI	(B2 − B4)/B3	*y* = −2.78 × *x* + 2.30	1.08	3.80	1.95
	Sentinel MSI	B1	*y* = −10.42 × *x* + 2.76	1.44	4.36	2.09

**Table 14:** The component matrix by SPSS.

	PC1	PC 2	PC 3	PC 4
pH	0.958	−0.023	−0.139	−0.016
DO	0.844	0.173	−0.293	0.034
Chl-a concentration	0.800	−0.283	0.267	0.044
WT	0.660	−0.471	0.216	−0.041
TN	0.573	0.427	0.183	−0.074
COD_Mn_	0.514	0.428	0.008	0.138
Conductivity	0.478	0.572	0.375	−0.039
Algal density	0.370	−0.483	0.358	0.131
TP	−0.365	0.452	0.322	0.187
Turbidity	−0.341	−0.057	0.818	0.004
NH_3_-N	−0.011	−0.040	−0.082	0.958
Variability (%)	35.569	13.341	11.870	9.103
Cumulative (%)	35.569	48.910	60.780	69.883

## Data Availability

The data presented in this study are available on request from the corresponding author.
